# A novel extracellular vesicles production system harnessing matrix homeostasis and macrophage reprogramming mitigates osteoarthritis

**DOI:** 10.1186/s12951-024-02324-8

**Published:** 2024-02-28

**Authors:** Tianqi Wang, Hongqi Zhao, Yi Zhang, Yanshi Liu, Jialin Liu, Ge Chen, Ke Duan, Zhong Li, Hoi Po James Hui, Jiyuan Yan

**Affiliations:** 1 Department of Orthopaedics, The Affiliated Hospital of Southwest Medical University, Luzhou, Sichuan 646000 China; 2 Departments of Orthopaedic Surgery, Yong Loo Lin School of Medicine, National University of Singapore, Singapore, 117597 Singapore; 3 Tissue Engineering Program, Yong Loo Lin School of Medicine, Life Sciences Institute, National University of Singapore, Singapore, 117597 Singapore; 4 School of Public Health, Southwest Medical University, Luzhou, 646000 Sichuan China; 5 Sichuan Provincial Laboratory of Orthopaedic Engineering, Luzhou, Sichuan 646000 China; 6 Department of Oral Implantology, The Affiliated Stomatology Hospital of Southwest Medical University, Luzhou, Sichuan 64600 China; 7 Department of Orthopedics, Tongji Hospital, Tongji Medical College, Huazhong University of Science and Technology, Wuhan, 430030 China

**Keywords:** Extracellular vesicles, Electromagnetic field, Matrix homeostasis, Macrophage, Reprogramming, Osteoarthritis

## Abstract

Osteoarthritis (OA) is a degenerative disease that significantly impairs quality of life. There is a pressing need for innovative OA therapies. While small extracellular vesicles (sEVs) show promising therapeutic effects against OA, their limited yield restricts clinical translation. Here, we devised a novel production system for sEVs that enhances both their yield and therapeutic properties. By stimulating mesenchymal stem cells (MSCs) using electromagnetic field (EMF) combined with ultrasmall superparamagnetic iron oxide (USPIO) particles, we procured an augmented yield of EMF-USPIO-sEVs. These vesicles not only activate anabolic pathways but also inhibit catabolic activities, and crucially, they promote M2 macrophage polarization, aiding cartilage regeneration. In an OA mouse model triggered by anterior cruciate ligament transection surgery, EMF-USPIO-sEVs reduced OA severity, and augmented matrix synthesis. Moreover, they decelerated OA progression through the microRNA-99b/MFG-E8/NF-κB signaling axis. Consequently, EMF-USPIO-sEVs present a potential therapeutic option for OA, acting by modulating matrix homeostasis and macrophage polarization.

**Supplementary Information:**

The online version contains supplementary material available at 10.1186/s12951-024-02324-8.

## Introduction

Osteoarthritis (OA) is a chronic, progressive disorder marked by cartilage destruction and secondary synovial inflammation [1]. This condition manifests as joint pain, dysfunction, and in severe cases, disability, critically impairing patient quality of [2]. Current therapeutic options are limited to medication and surgery. Pharmacological interventions may provide temporary pain relief and manage symptoms. However, they do not halt or slow cartilage destruction [3] owing to limited bioavailability, reduced stability, and swift joint clearance [4]. In contrast, joint replacement surgery, while effective in halting disease progression [5], is associated with high costs and an elevated risk of surgical complications [6]. Given these challenges, there’s a pressing need to innovate therapeutic methods for early-stage OA, necessitating a more profound grasp of OA’s pathogenic underpinnings.

The etiologies and pathogenic mechanisms underlying OA onset and progression are complex. Imbalances in extracellular matrix (ECM) homeostasis is widely recognized as a trigger for cartilage destruction [7]. Under these conditions, numerous matrix-degrading enzymes are substantially upregulated [8]. Additionally, substantial evidence has highlighted a vital part of synovial macrophages in OA progression [9]. Macrophages are categorized as pro-inflammatory M1 and anti-inflammatory M2 macrophages [10]. Imbalances in M1/M2 macrophage ratio are strongly associated with OA severeness [11]. M1 macrophages predominate the early inflammation stage, secrete pro-inflammatory cytokines and finally accelerate cartilage degeneration [12]. Conversely, M2 macrophages release anti-inflammatory cytokines that aid in resolving inflammation [13].

Small extracellular vesicles (sEVs) have gained attention as potential cell-free therapeutic agents for degenerative diseases due to their abilities in immune modulation and tissue regeneration [14]. By transferring bioactive components, such as lipids, microRNAs (miRNAs), lncRNAs, and specific proteins, sEVs facilitate intercellular communication and modulate recipient cell functions [15]. Increasing evidence indicates that mesenchymal stem cell (MSC)-derived sEVs (MSC-sEVs) have substantial therapeutic effects on OA [16]. For instance, human MSC-sEVs protect the cartilage from degeneration [17]. Additionally, in studies involving collagen-induced OA models, sEVs from synovium-derived MSCs ameliorated OA symptoms [18]. However, clinically applying sEVs is impeded by their limited production rates. The average sEVs yield from one million cells is 1–4 µg daily, falling short of the requirement for clinical trials (100–500 µg per patient) [19].

In this study, a novel sEVs production system was developed to optimize the yield and cargo content of sEVs, enhancing their therapeutic efficacy. This approach hinged on stimulating BMSCs with electromagnetic field (EMF) and ultrasmall superparamagnetic iron oxide (USPIO) particles, thereby generating enriched sEVs. EMF has emerged as a non-invasive, non-pharmacological, and conservative OA treatment. In vitro research have shown that EMF promotes chondrocyte proliferation, synthetic activity, and phenotypic maturation while reducing pro-inflammatory cytokine levels in human chondrocytes considerably [20]. Numerous multicenter, randomized, double-blind clinical trials have yielded promising results [21]. Consequently, European League Against Rheumatism ranked EMF therapy for OA as a 1B evidence and assigned a B rating for its recommendation strength. USPIO, a type of cationic magnetic nanoparticle, has been extensively studied for intra-articular treatment [22]. These nanoparticles can be rapidly adsorbed onto the cartilage owing to electrostatic interactions with the anionic cartilage-ECM, preventing their swift clearance and resulting in deep penetration and prolonged residence.

**Scheme 1:**
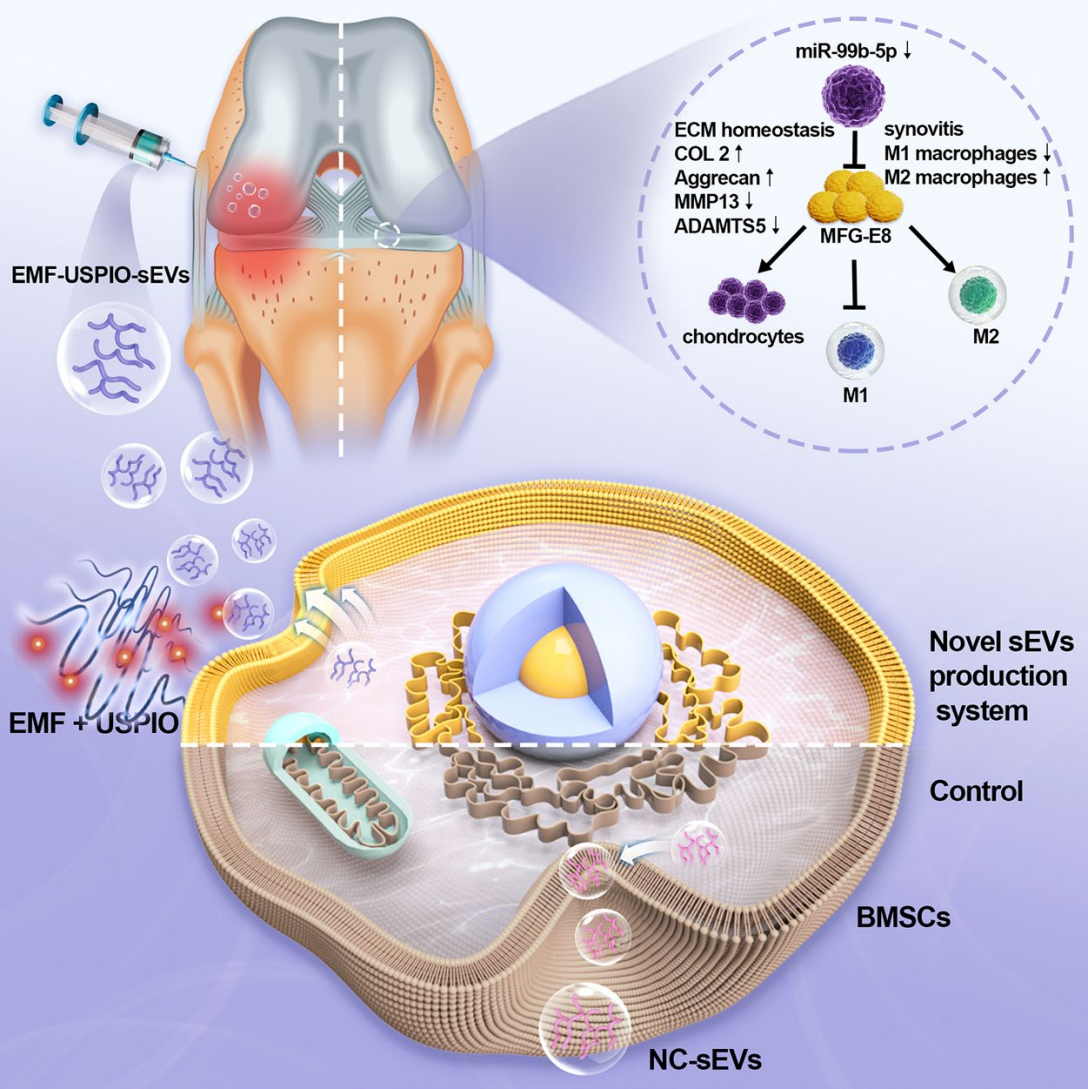
Summary of a novel small extracellular vesicles production system for osteoarthritis treatment

We investigated the therapeutic efficacy and mechanism of action of sEVs derived from BMSCs pre-stimulated with an EMF and USPIO (EMF-USPIO-sEVs) to determine whether the cell-free therapy could be applied to OA (Scheme 1). Our findings introduce a novel sEVs-based approach for treating OA, markedly boosting both the yield and cargo content of sEVs. Such enhancements not only bolster the promise for OA therapy but also broaden the potential applicability of sEVs in treating other diseases and conditions.

## Results

### Characteristics of EMF and USPIO

**Fig. 1:**
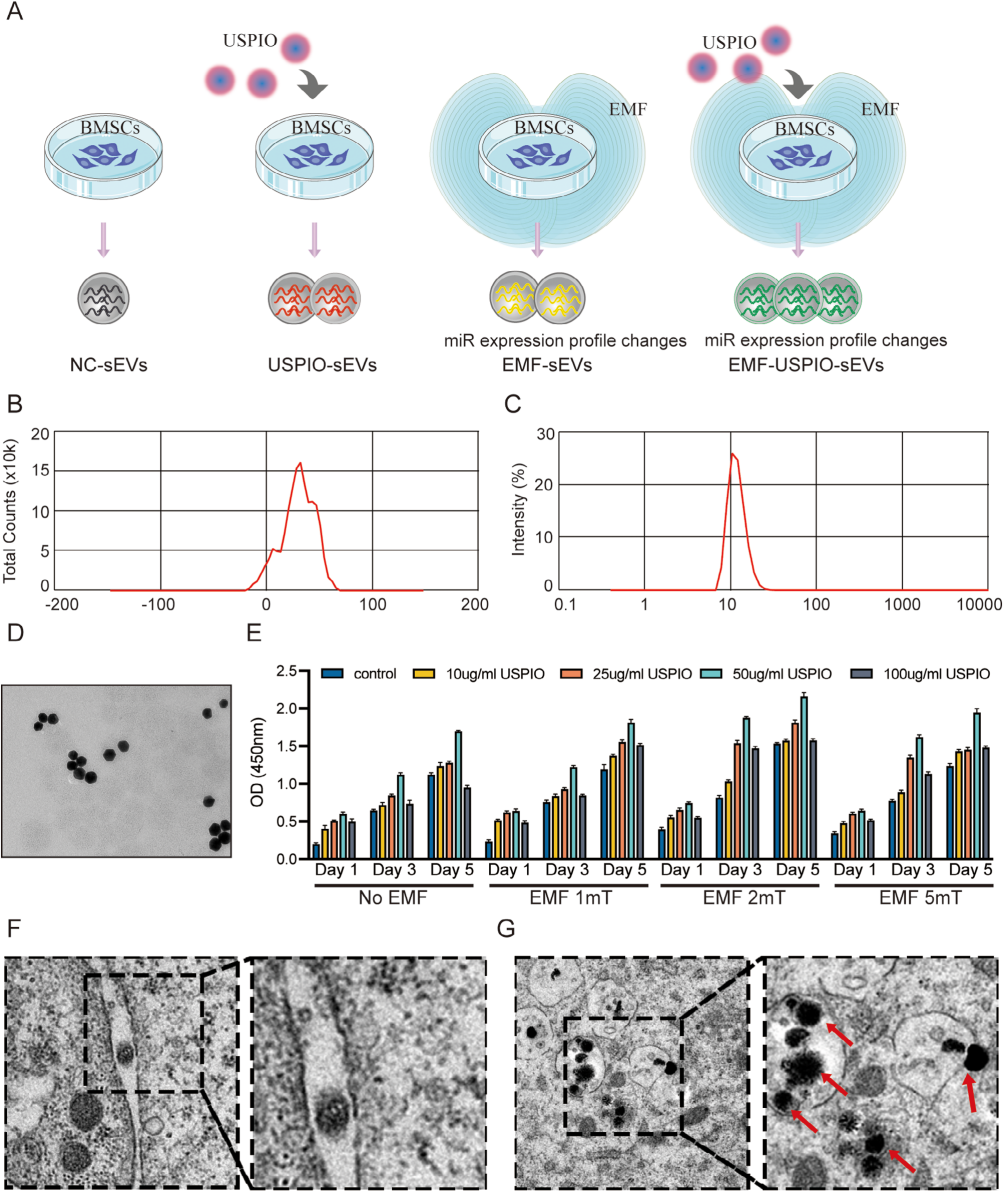
Production of four distinct sEVs: NC-sEVs, USPIO-sEVs, EMF-sEVs, and EMF-USPIO-sEVs. (**A**) Schematic representation of an innovative sEVs production system employing EMF and USPIO. **B-D**: Evaluations of the USPIO include zeta potential (**B**), diameter measurements (**C**), and morphological analysis (**D**). (**E**) Results from the Cell Counting Kit-8 assay for BMSCs subjected to various conditions, with USPIO concentrations at 0, 10, 25, 50, and 100 µg/mL and EMF intensities of 0, 1, 2, or 5 mT. (**F**) TEM images of BMSCs without USPIO treatment. The right image is an enlargement of the marked box in the left image. (**G**) TEM images of BMSCs with USPIO treatment. The black dotted box indicates USPIO internalization by BMSCs, and the red arrows point to the USPIO

This innovative sEVs production system integrates both EMF and USPIO components. The superparamagnetic nature of USPIO enables efficient responsiveness to EMF stimulation, facilitating the rapid translation of electromagnetic signals into biological responses. Figure 1A is a schematic diagram of the sEVs under four distinct production conditions: NC-sEVs, USPIO-sEVs, EMF-sEVs, and EMF-USPIO-sEVs. The USPIO surface was positively charged with an average zeta potential and diameter of + 29.0 mV (Fig. 1B) and 12.15 nm (Fig. 1C), respectively. Further, the USPIO particles were found to be spherical in shape with a diameter of approximately 10 nm (Fig. 1D). TEM images revealed that BMSCs internalized USPIO, which were uniformly distributed in the cytoplasm (Fig. 1F-G). From a functional perspective, the CCK-8 assay revealed that combining 2 mT EMF and 50 g/mL USPIO provided optimal conditions for BMSC growth and proliferation (Fig. 1E). Consequently, 2 mT EMF and 50 µg/mL USPIO were the adopted for subsequent experiments.

Next, SEM-EDX mapping was used to verify the USPIO composition. EDX mapping revealed a composite of carbon (C), nitrogen (N), oxygen (O), iron (Fe), and titanium (Ti) and elemental distribution within USPIO (Supplementary Fig. 1A). Additionally, the EDX spectrum highlighted the elements detected and their relative contents in USPIO (Supplementary Fig. 1B and Supplementary Table 1).

### Characteristics and internalization of sEVs

**Fig. 2:**
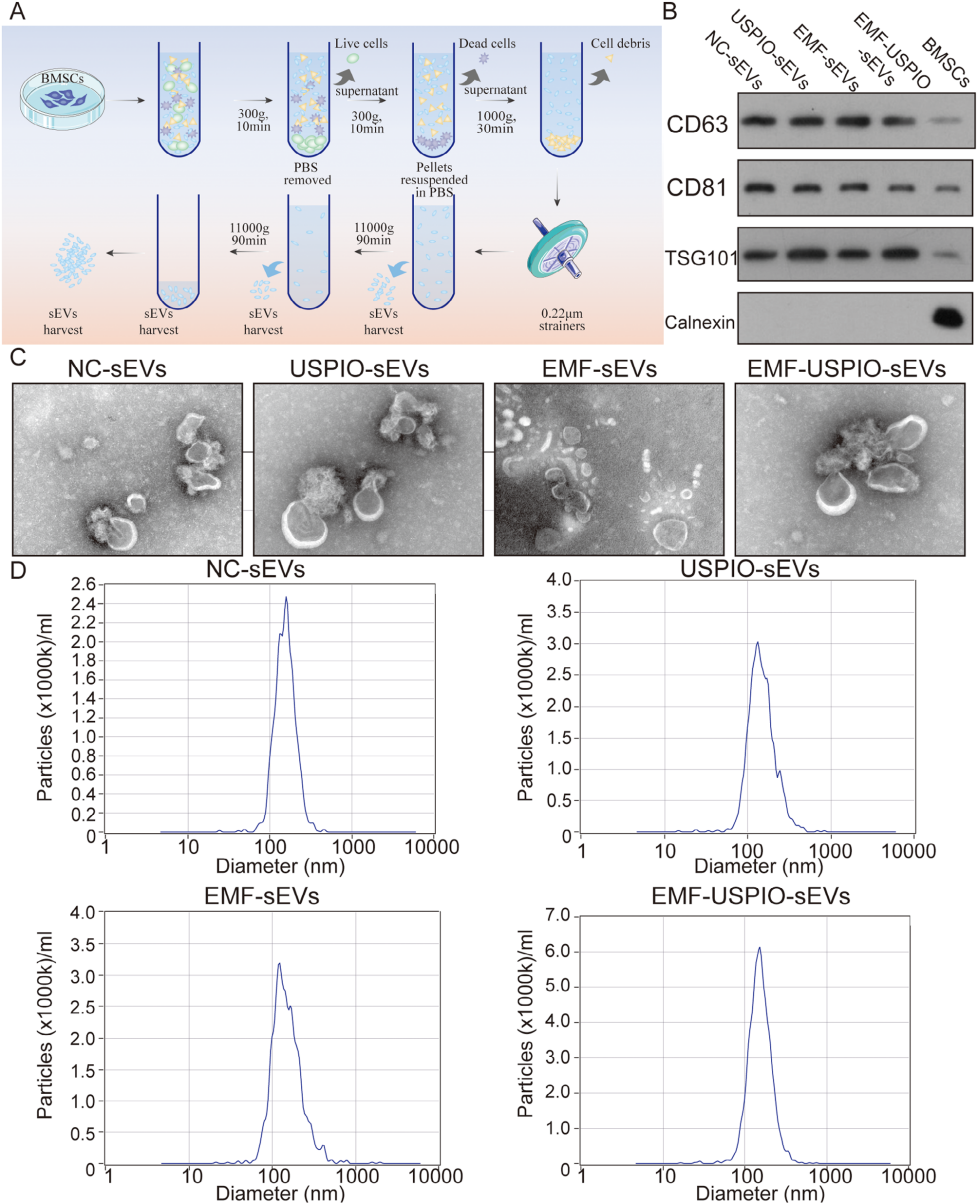
Characterization of the four sEVs. (**A**) Schematic representation of the sEVs isolation process using differential ultracentrifugation. (**B**) Western blot analysis for sEV-specific marker proteins, namely CD63, CD81, and TSG101, as well as the negative marker, Calnexin. (**C**) Morphology of the four sEVs types observed under TEM. (**D**) NTA analysis of the four sEVs types revealed similar size ranges

Subsequently, we identified four distinct types of sEVs. Figure 2A is a schematic diagram of the sEVs extraction yield using differential ultracentrifugation. Western blotting verified the expression of sEVs-specific markers (CD63, CD81, and TSG101) in all sEVs samples. In contrast, the negative marker, Calnexin, was absent (Fig. 2B). TEM images demonstrated that all four types of sEVs exhibited similar cup-shaped morphologies (Fig. 2C). Intriguingly, no significant differences existed in their sizes, shapes, or markers. NTA analysis indicated that the particle sizes of all four types of sEVs were predominantly 120–160 nm (Fig. 2D). Notably, this analysis also showed that the sEVs yield was markedly elevated in the EMF-sEVs and EMF-USPIO-sEVs groups than that in the NC-sEVs group (Supplementary Fig. 2A). In addition, nano-flow cytometry confirmed that all four groups expressed sEVs-specific markers (CD9, CD63, and CD81) (Supplementary Fig. 3A). A more comprehensive analysis revealed that the EMF-USPIO-sEVs group had the highest positive markers (Supplementary Fig. 2B-D and Supplementary Fig. 3A). Supplementary Fig. 3B illustrated efficient sEVs uptake by primary chondrocytes at different time points (24 and 36 h).

### EMF-USPIO-sEVs restored chondrocyte homeostasis in vitro

**Fig. 3:**
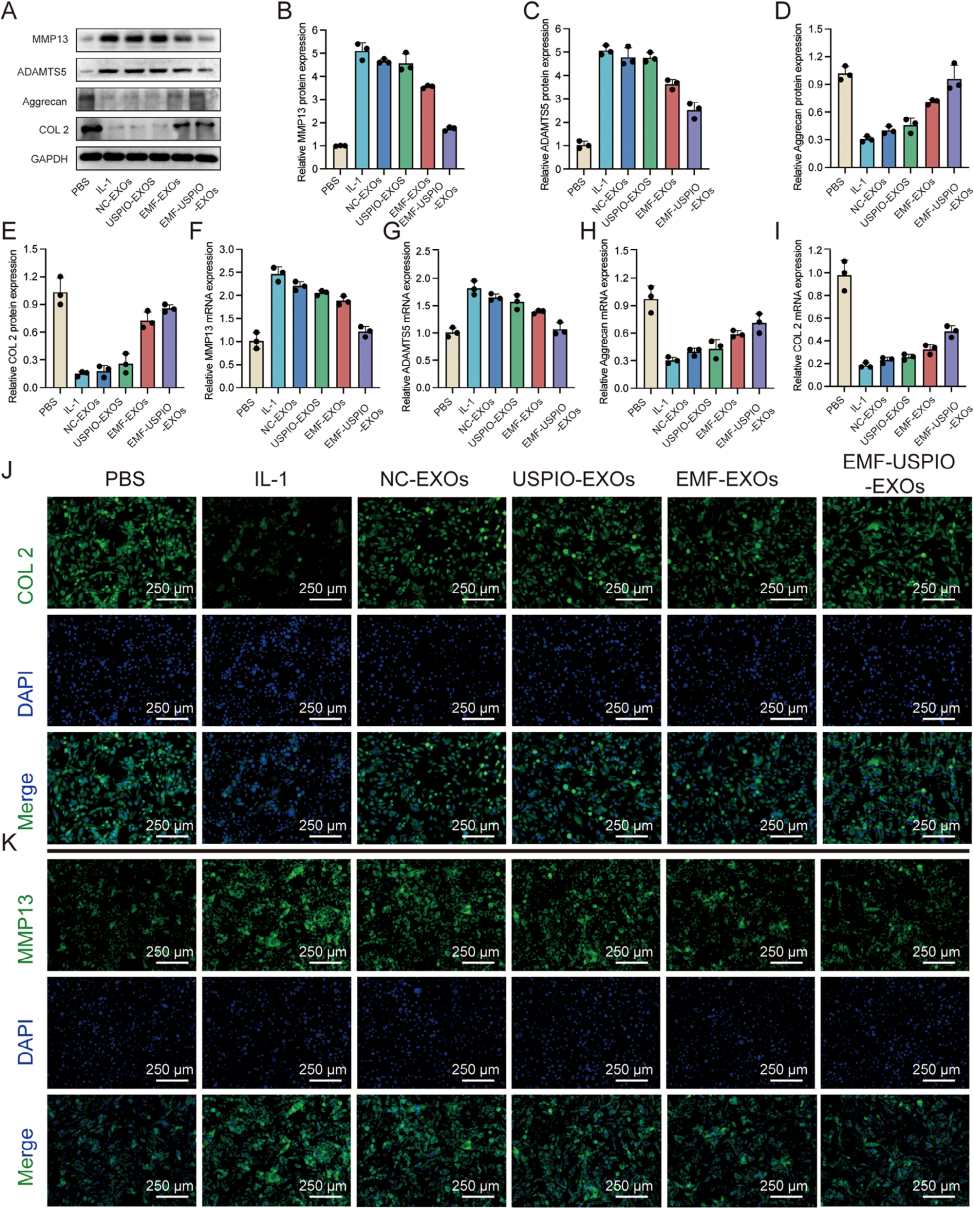
EMF-USPIO-sEVs restored chondrocyte homeostasis. **A-E**: Western Blotting bands (**A**) of MMP13, ADAMTS5, Aggrecan, and COL 2, and corresponding semi-quantifications of the expression levels of MMP13 (**B**), ADAMTS5 (**C**), Aggrecan (**D**) and COL 2 (**E**). **F-I**: Relative mRNA expression levels of MMP13 (**F**), ADAMTS5 (**G**), Aggrecan (**H**), and COL 2 (**I**). **J-K**: Immunofluorescence images of COL 2 (**J**) and MMP13 (**K**) following various treatments (PBS, IL-1, NC-sEVs, USPIO-sEVs, EMF-sEVs, and EMF-USPIO-sEVs). COL2/MMP13 were labeled with FITC (green), and nuclei were labeled with DAPI (blue). *p < 0.05, **p < 0.01, ***p < 0.001 compared to PBS group; #*p* < 0.05, ##*p* < 0.01 compared to EMF-sEVs group

Owing to the critical role of ECM homeostasis in OA cartilage repair and regeneration, western blotting and qRT-PCR were employed to directly evaluate the protein and mRNA levels of catabolic (MMP13 and ADAMTS5) and anti-catabolic (Aggrecan and COL 2) molecules, respectively. IL-1β is a prevalent cytokine that can induce an OA-like microenvironment in vitro. NC-sEVs and USPIO-sEVs did not modulate chondrocyte metabolism but partially reversed the imbalance in metabolic homeostasis. EMF-USPIO-sEVs exhibited increased expression of anti-catabolic markers and decreased catabolic marker expression (Fig. 3A-I). Microscopic observations (Fig. 3J-K and Supplementary Fig. 4A-B) were consistent with the western blotting and PCR results, demonstrating that EMF-USPIO-sEVs restored chondrocyte homeostasis more effectively than the original sEVs. Briefly, EMF-USPIO-sEVs promoted anabolism and inhibited, indicating that they could maintain matrix homeostasis and promote cartilage repair and regeneration.

### EMF-USPIO-sEVs enhanced M2 macrophage polarization in vitro

**Fig. 4:**
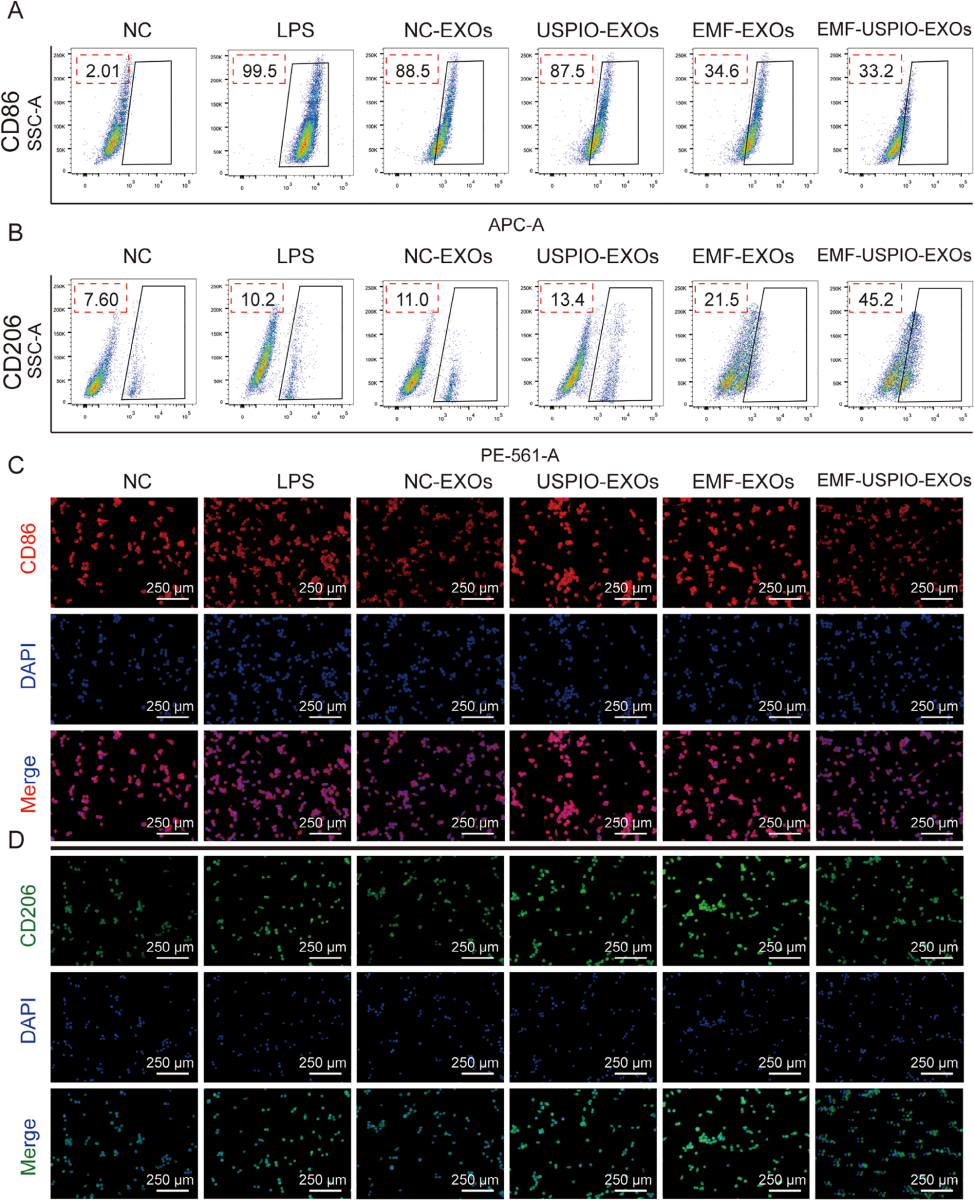
EMF-USPIO-sEVs promoted M2 macrophage polarization. (**A**) EMF-USPIO-sEVs downregulated the proportion of CD206 + macrophages. (**B**) EMF-USPIO-sEVs upregulated the proportion of CD86 + macrophages. **C-D**: Immunofluorescence images of CD86 (**C**) and CD206 (**D**) following various treatments (PBS, IL-1, NC-sEVs, USPIO-sEVs, EMF-sEVs, and EMF-USPIO-sEVs). CD86 was stained with Cy3, exhibiting a red fluorescence; CD206 was stained with FITC, producing a green fluorescence; and nuclei were stained with DAPI, resulting in a blue fluorescence

The polarized phenotype of synovial macrophages is associated with OA pathogenesis and progression. The immunomodulatory effects of sEVs on macrophages were investigated using flow cytometry and immunofluorescence. LPS treatment induced M1 polarization, as indicated by the elevated CD86 expression (Fig. 4A and C). Flow cytometry revealed that EMF-sEVs and EMF-USPIO-sEVs effectively induced the M2 polarization, as demonstrated by the increased ratio of CD206 + and decreased ratio of CD86 + macrophages, respectively. EMF-USPIO-sEVs were more effective than EMF-sEVs (Fig. 4A-B and Supplementary Fig. 4C-D). Additionally, cell immunofluorescence supported these findings (Fig. 4c-d and Supplementary 4E-F). Thus, EMF-USPIO-sEVs exhibited superior immunosuppressive effects by inducing the M2 polarization, potentially mitigating OA progression and preserving cartilage integrity.

### EMF-USPIO-sEVs suppressed OA progression in vivo

**Fig. 5:**
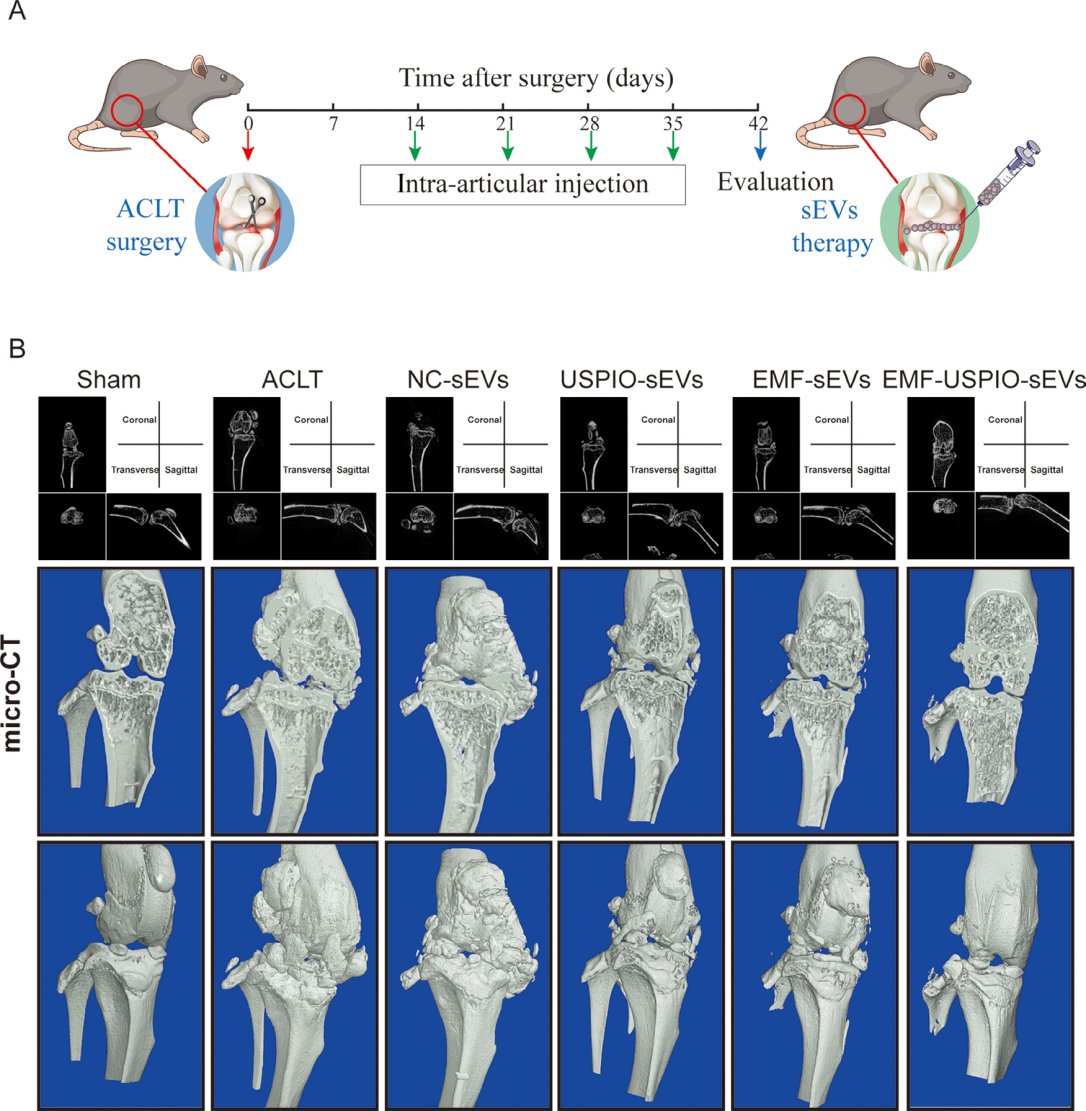
micro-CT assessment of cartilage repair. (**A**) Schematic illustration of intra-articular injection of EMF-USPIO-sEVs in an ACLT mouse model. (**B**) Three-dimensional reconstructions generated from micro-CT, illustrating the state of cartilage repair following various treatments (Sham, ACLT, NC-sEVs, USPIO-sEVs, EMF-sEVs, and EMF-USPIO-sEVs)

An ACLT-induced OA mouse model was used to assess the protective effects of sEVs in vivo. Throughout the study, no mouse exhibited signs of anorexia, diarrhea, lethargy, or infection. Two weeks after the ACLT surgery, 10 µL of PBS, NC-sEVs, USPIO-sEVs, EMF-sEVs, or EMF-USPIO-sEVs was administered weekly into the right articular cavity. Four weeks later, the joint specimens were collected (Fig. 5A). Micro-CT analysis and three-dimensional (3D) modeling were used to evaluate osteophyte formation. While the total osteophyte volume showed a marked increase in the ACLT and NC-sEVs groups comparing with Sham groups (healthy control), there was a decrease observed in the USPIO-sEVs, EMF-sEVs, and EMF-USPIO-sEVs groups comparing with Sham groups (healthy control) (Fig. 5B and Supplementary Fig. 5B).

**Fig. 6:**
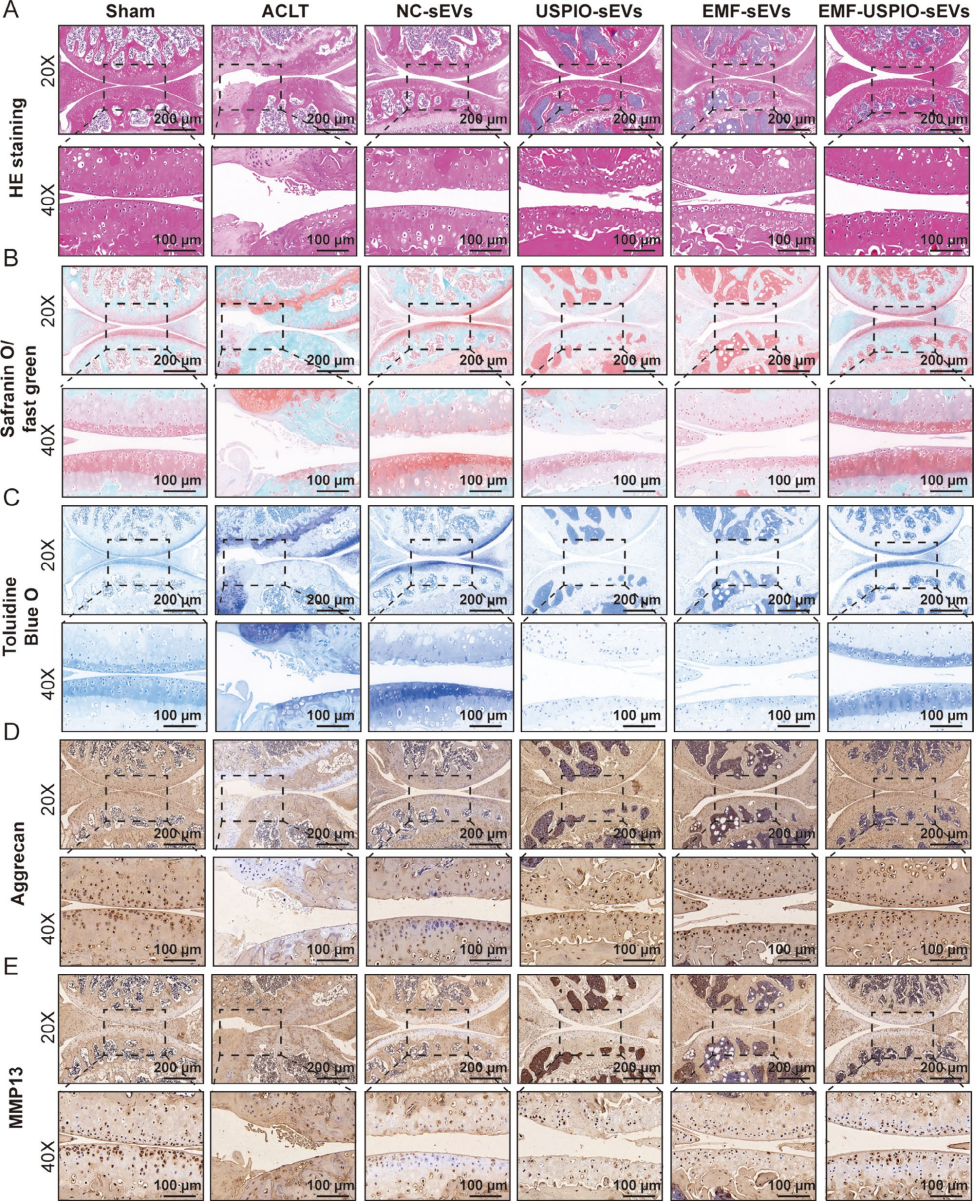
Histological verifications (HE, SO-FG, TB staining, and immunohistochemical staining) of cartilage repair. **A-C**: HE (**A**), SO-FG (**B**), and TB (**C**) staining images detecting the cartilage repair condition. **D-E**: Immunohistochemical staining of the anti-catabolic marker (Aggrecan) (**D**) and catabolic marker (MMP13) (**E**)

H&E, SO-FG, and TB staining were performed to assess cartilage damage and proteoglycan depletion. HE staining illustrated the smoothest and roughest surfaces in the sham and ACLT groups, respectively (Fig. 6A). Cartilage surfaces in the NC-sEVs and USPIO-sEVs groups were rough. In contrast, those in the EMF-sEVs and EMF-USPIO-sEVs groups were smooth (Fig. 6A). This suggested that EMF-sEVs and EMF-USPIO-sEVs exhibited enhanced therapeutic effects. EMF-USPIO-sEVs had the most substantial impact on cartilage repair (Fig. 6A). Cartilage polysaccharides were stained red using safranin O. Bone tissue was stained green using fast green. A deeper red color indicated a higher polysaccharide concentration. Polysaccharides were nearly undetectable in the ACLT, NC-sEVs, and USPIO-sEVs groups (Fig. 6B). However, their levels were notably higher in the EMF-sEVs and EMF-USPIO-sEVs groups than in the ACLT group, indicating an increase in polysaccharide content as cartilage repair progressed (Fig. 6B). Moreover, EMF-USPIO-EVs enhanced polysaccharide levels more significantly than EMF-EVs (Fig. 6B). TB staining yielded similar results with EMF-USPIO-sEVs, which exhibited the most significant effect on cartilage repair (Fig. 6C). OARSI scoring was employed to assess cartilage damage severity, and EMF-USPIO-sEVs substantially decreased the severity of cartilage lesions (Supplementary Fig. 5A).

To further explore the effects of EMF-USPIO-sEVs treatment on ECM homeostasis following ACLT surgery in mice, immunohistochemical staining was performed to examine aggrecan and MMP13 expression levels. EMF-USPIO-sEVs significantly reversed ACLT-induced downregulation of aggrecan and upregulation of MMP13 in chondrocytes (Fig. 6D-E and Supplementary Fig. 5C–D). Overall, EMF-USPIO-sEVs prevented cartilage destruction considerably.

### miRNA profile and bioinformatics of EMF-USPIO-sEVs

**Fig. 7:**
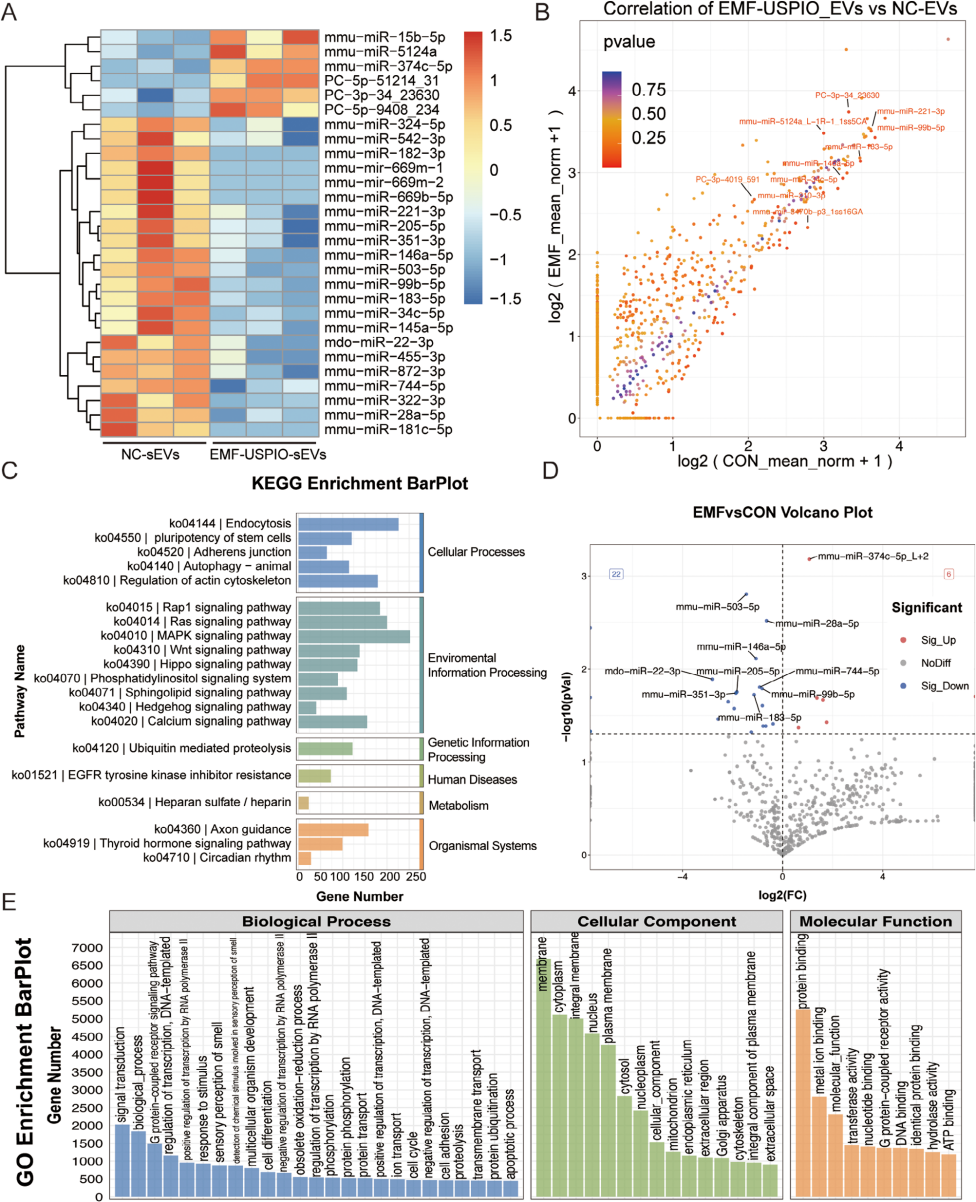
miRNA sequencing and bioinformatics analysis. (**A**) Heatmap illustrating differentially expressed miRNAs between NC-sEVs and EMF-USPIO-sEVs. (**B**) Scatter plot depicting the expression profile and global distribution of 28 differentially expressed miRNAs between NC-sEVs and EMF-USPIO-sEVs. (**C**) KEGG pathway enrichment analysis reveals several signaling pathways associated with OA progression. (**D**) Volcano plot highlights mRNAs exhibiting a ≥ 2-fold differential expression between NC-sEVs and EMF-USPIO-sEVs. Green represents downregulated mRNAs, while red denotes upregulated ones. (**E**) GO enrichment analysis of differentially expressed miRNAs

miRNA sequencing analysis was initially conducted on the NC-sEVs and EMF-USPIO-sEVs groups to uncover the potential molecular mechanism of action of EMF-USPIO-sEVs. The heatmap revealed that the expression levels of 6 miRNAs increased and those of 22 miRNAs decreased in the EMF-USPIO-sEVs group compared to NC-sEVs group (Fig. 7A). Scatter (Fig. 7B) and volcano plots (Fig. 7D) were used to depict the expression profiles and global distributions of the 28 differentially expressed miRNAs, respectively. The analysis of KEGG pathway enrichment revealed numerous signaling pathways that are closely linked to the progression of OA, such as MAPK and Wnt (Fig. 7C). The analysis of gene ontology showed significant participation in the biological process category, specifically in ‘signal transduction’ and ‘positive regulation of transcription by RNA polymerase II promoter’. In the cellular component category, the involvement was observed in ‘membrane’ and ‘cytoplasm’. Lastly, within the molecular function category, the significant activities were found in ‘protein binding’ and ‘transferase activity’ (Fig. 7E). Overall, these findings underscore the importance of miRNAs in halting OA progression.

### miR-99b-5p was downregulated in EMF-USIPO-sEVs

**Fig. 8:**
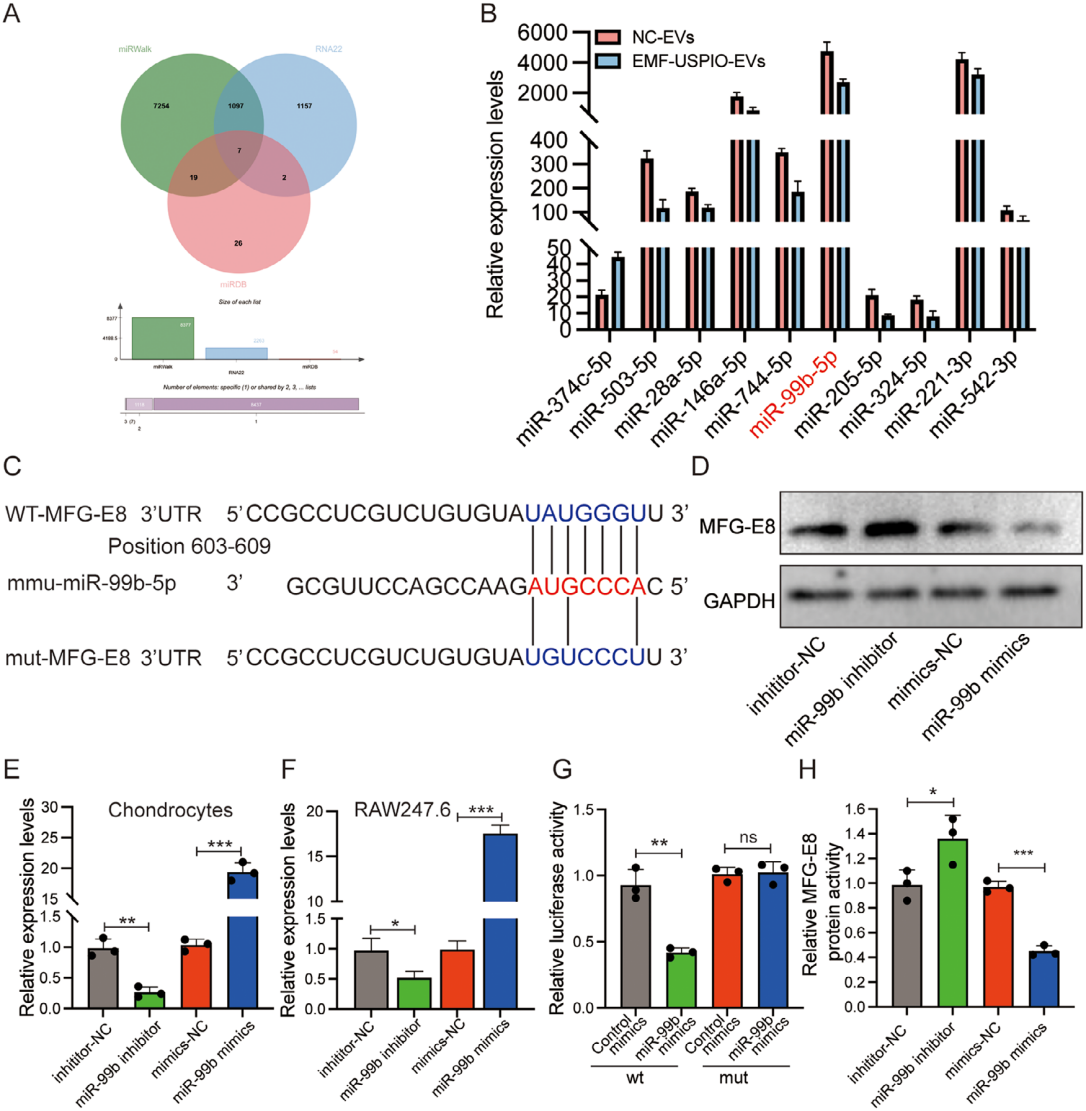
miR-99b-5p derived from EMF-USPIO-sEVs regulated MFG-E8 expression. (**A**) Venn diagram displaying the potential target genes of miR-99b-5p, as predicted by miRWalk, RNA22, and miRDB databases. (**B**) Comparison of the top ten differentially expressed miRNAs (miR-374c-5p, miR-503-5p, miR-28a-5p, miR-146a-5p, miR-744-5p, miR-99b-5p, miR-205-5p, miR-324-5p, miR-221-3p, and miR-542-3p) between NC-sEVs and EMF-USPIO-sEVs using qRT-PCR. (**C**) miR-99b-5p binding sequence in the 3′-UTR of MFG-E8. **D**, **H**: Protein expression of MFG-E8 after cell transfection with miR-99b-5p mimics, miR-99b-5p inhibitor, and their respective NCs and semi-quantification. **E-F**: Transfection efficiency confirmation of miR-99b-5p in chondrocytes (**E**) and RAW246.7 (**F**). (**G**) Luciferase readings were obtained from either wt or mut MFG-E8 3′-UTR when co-transfected with control mimics or miR-99b-5p mimics. Transfection with miR-99b-5p mimics led to a reduction in luciferase activity relative to the control mimics, substantiating that MFG-E8 is a direct target of miR-99b-5p

Given the miRNA sequencing data, the top 10 differentially expressed miRNAs (miR-374c-5p, miR-503-5p, miR-28a-5p, miR-146a-5p, miR-744-5p, miR-99b-5p, miR-205-5p, miR-324-5p, miR-221-3p, and miR-542-3p) were selected, and their expression was further validated using qRT-PCR. As depicted in Fig. 8B, miR-99b exhibited the highest expression level and was significantly downregulated in EMF-USPIO-sEVs than in NC-sEVs. Based on the miRNA sequencing results, qRT-PCR data, gene enrichment analysis (including KEGG and GO), and previous literature, miR-99b-5p appears to affect cartilage repair negatively. Hence, we examined the association of miR-99b-5p with cartilage repair enhancement using EMF-USPIO-sEVs. The miRWalk, RNA22, and miRDB databases have predicted the potential target genes of miR-99b-5p, as shown in Fig. 8A. Chondrocytes (Fig. 8E) and RAW246.7 (Fig. 8F) were transfected with inhibitors or mimics of miR-99b-5p, as well as their corresponding controls. The validation of transfection efficiency was performed by employing qRT-PCR.

### miR-99b-5p modulated MFG-E8 expression

To verify predicted direct interaction between miR-99b-5p and its target gene MFG-E8, luciferase reporter assays were performed with plasmids that contained either the wild-type or the mutated 3′-UTR of MFG-E8, as suggested by online databases (Fig. 8C). Transfection with miR-99b-5p mimics leads to a decrease in luciferase activity when compared to control mimics (Fig. 8G). In line with previous assay findings, transfection with miR-99b-5p mimics notably reduced MFG-E8 levels in chondrocytes, whereas treatment with the miR-99b-5p inhibitor produced the opposite outcome (Fig. 8D and H).

### miR-99b-5p exacerbated OA through the MFG-E8/NF- κB pathway

**Fig. 9:**
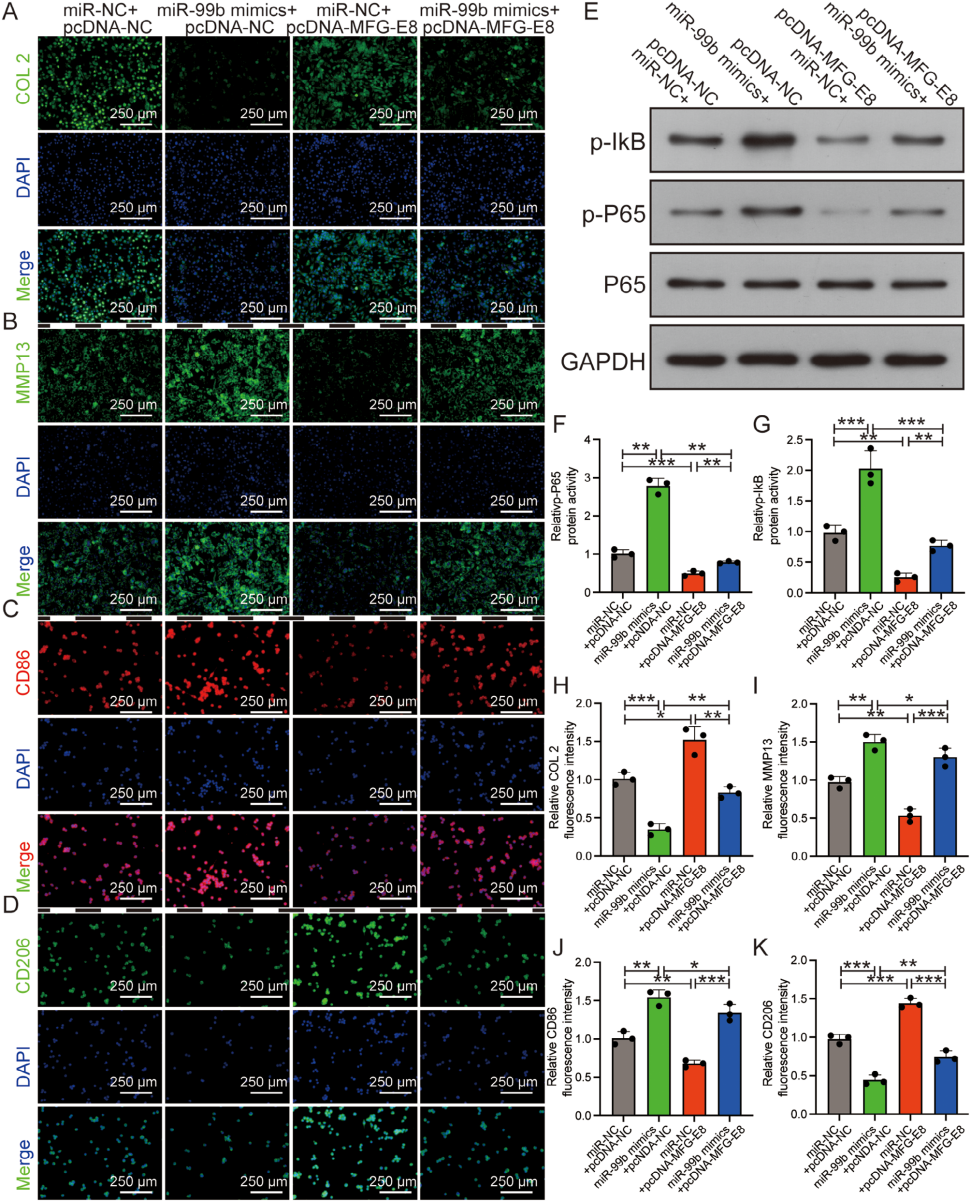
miR-99b-5p exacerbated OA through the MFG-E8/NF- κB pathway. **A-D**, **H-K**: Immunofluorescence images of COL 2 (**A**), MMP13 (**B**), CD86 (**C**), and CD206 (**D**) following various treatments (miR-NC + pcDNA-NC, miR-99b-5p + pcDNA-NC, miR-NC + pcDNA-MFG-E8, and miR-99b-5p + pcDNA-MFG-E8); corresponding semi-quantifications of fluorescence intensity of COL 2 (**H**), MMP13 (**I**), CD86 (**J**) and CD206 (**K**). COL 2, MMP13, and CD206 were labeled with FITC (green), CD86 was labeled with Cy3 (red), and nuclei were labeled with DAPI (blue). E-G: Western Blotting bands (**E**) of p-P65 and p-IkB and corresponding semi-quantifications of the expression levels of p-P65 (**F**) and p-IkB (**G**). *p < 0.05, **p < 0.01, ***p < 0.001

Further investigations into the relationship between miR-99b-5p and MFG-E8 were carried out through a sequence of rescue experiments. Chondrocytes co-transfected with miR-99b-5p mimics and pcDNA-NC exhibited a notable reduction in the fluorescence intensity of advantageous OA markers (COL2 and CD206). Contrastingly, COL2 and CD206 expression levels were the highest when co-transfected with miR-NC mimics and pcDNA-MFG-E8 (Fig. 9A, D and H, and 9K). The opposite was observed for the harmful OA markers (MMP13 and CD86) (Fig. 9B-C and I-J).

Next, we aimed to identify the miR-99b-5p-mediated pathways contributing to chondrocyte degeneration and macrophage reprogramming during OA. Interestingly, our findings indicate that MFG-E8 restored p65 phosphorylation in the NF-κB signaling pathway. In contrast, miR-99b-5p treatment significantly increased p65 phosphorylation (Fig. 9E-G). This series of rescue experiments revealed that miR-99b-5p downregulation in EMF-USPIO-sEVs inhibited OA progression through the MFG-E8 axis.

## Discussion

MSC-sEVs have attracted considerable attention owing to their therapeutic potential in treating OA. These nano-sized vesicles can transport various bioactive molecules to injured chondrocytes, enhancing their functions. MSC-sEVs can treat OA through several mechanisms, including cartilage homeostasis regulation and inflammatory response modulation [24]. ECM homeostasis balance is disrupted by the increased and decreased expression of degrading and synthesizing enzymes, respectively, resulting in cartilage degradation [25]. This study examined the impact of EMF-USPIO-sEVs on chondrocyte homeostasis under IL-1β treatment. Our results revealed that EMF-USPIO-sEVs effectively suppressed IL-1β-induced ADAMTS-5 and MMP13 expression while enhancing aggrecan and COL 2 expression in chondrocytes, consistent with previous studies [24c]. These findings indicate that EMF-USPIO-sEVs helped maintain ECM homeostasis.

Furthermore, macrophages are vital for OA progression. Prior studies have demonstrated that MSC-sEVs promote M2 macrophage polarization, reducing pro-inflammatory cytokine expression [24b, 26]. Our findings suggest that EMF-USPIO-sEVs promote macrophage polarization toward the M2 phenotype. Consequently, these vesicles exhibit immunosuppressive properties that may mitigate OA progression and prevent cartilage degeneration. The ability of EMF-USPIO-sEVs to regulate ECM homeostasis and macrophage polarization emphasizes their therapeutic potential in halting OA progression. However, the low sEVs yield limits their clinical application. We developed a novel sEVs production system in OA treatment, as described earlier, to overcome this limitation. This system aims to enhance the therapeutic potential of sEVs and improve their applicability in OA treatment.

EMF therapy, a non-invasive and non-thermal treatment, is widely employed to manage OA-associated joint pain, indicating a promising theoretical basis for clinical application [27]. Previous studies have revealed that EMF can restrict the destructive impacts of pro-inflammatory cytokines on hyaline cartilage, indicating their potential to reduce inflammation and promote soft tissue repair [28]. Recently, cationic nanocarriers have garnered significant attention owing to their application in intra-articular drug delivery [22, 29]. These carriers quickly adsorb onto the cartilage with a moderate affinity for anionic components within the cartilage via electrostatic interactions. Rapid adsorption helps prevent clearance from the joint. In contrast, moderate binding allows deep penetration and prolonged retention in the cartilage. Motivated by this cationic nanocarrier approach, we selected USPIO, a cationic magnetic nanoparticle, to respond to EMF stimulation and boost sEVs yield and cargo. Our results revealed that MSCs-sEVs treated with EMF and USPIO effectively restored chondrocyte homeostasis and enhanced M2 macrophage polarization compared with sEVs from untreated MSCs (NC-sEVs). In vitro and in vivo findings revealed that EMF-USPIO-sEVs mitigated OA progression more effectively than untreated control sEVs (NC-sEVs). These findings underscore the potential of this innovative approach for developing more effective OA therapies.

Recently, nucleic acids, including mRNAs, non-coding RNAs, and miRNAs, have been identified in sEVs. MiRNAs are key sEVs components critical to OA progression by regulating inflammatory response, chondrocyte survival, and ECM deposition [30]. These miRNAs transported by sEVs can be internalized by recipient cells and consequently exert significant biological functions [31]. In this study, we discovered a substantial decrease in miR-99b-5p in sEVs released by MSCs treated with EMF and USPIO. Additionally, we demonstrated that chondrocytes and RAW246.7 cells could internalize EMF-USPIO-sEVs, and miR-99b-5p interacted with the 3′-UTR of MFG-E8, leading to direct suppression. This observation provides further insights into the molecular mechanisms by which EMF-USPIO-sEVs may contribute to alleviating OA progression and the potential therapeutic applications of these vesicles.

MFG-E8 is a widely expressed glycoprotein that is crucial to inflammation [32]. In a study by Hansen et al., recombinant MFG-E8 resulted in a notable decrease in the expression of inflammatory factors in neonatal sepsis lung injury [33]. An additional significant discovery regarding MFG-E8 is its capacity to regulate macrophages in the direction of the M2 macrophage subtype [34]. In our study, we confirmed that the exogenous administration of MFG-E8 enhanced M2 macrophage polarization and restored the ECM during OA progression. More importantly, MFG-E8 significantly counteracted the miR-99b-5p mimic-induced ECM imbalance and M1 macrophage polarization, suggesting that the inhibition of MFG-E8 by miR-99b-5p might impact the OA progression. These findings shed light on the potential molecular mechanisms underlying OA and possible therapeutic targets for its treatment.

Emerging findings have highlighted the vital importance of the NF-κB pathway in osteoarthritis. Imbalanced activation of this pathway promotes the transcription of catabolic genes and triggers the release of inflammatory mediators through a loop of positive feedback, worsening the deterioration of cartilage [35]. In synovial tissues, NF-κB expression increases ten-fold during early OA, producing excessive inflammatory cytokines [36]. New research has additionally uncovered a robust connection between the NF-κB pathway and increased polarization of M1 macrophages [37]. Our study further validated NF-κB signaling’s involvement in OA-associated phenotypes.

Thus, the EMF-USPIO-sEVs investigated in this study mitigated OA through a miR-99b-5p/MFG-E8/B-dependent mechanism. These findings deepen our understanding of the molecular mechanisms that drive OA progression and offer potential therapeutic targets for developing novel treatments.

This study underscores the therapeutic potential of EMF-USPIO-sEVs, which reestablish chondrocyte homeostasis, promote M2 macrophage polarization, and serve as a biological vector for delivering decreased miR-99b-5p into recipient cells. However, this study has several limitations. First, additional research is required to elucidate how EMF and USPIO stimulate BMSCs to yield more sEVs containing decreased miR-99b-5p levels compared with unstimulated BMSCs, including the identification of upstream genes that regulate miR-99b-5p expression. Given the limitations of the mouse OA model, further investigations using larger animals are necessary to validate the therapeutic impact of EMF-USPIO-sEVs on OA. Addressing these limitations in future studies will facilitate advancements in OA therapy.

## Materials and methods

### Cell culture and treatment

BMSCs were obtained from Cyagen (Guangzhou, China) and cultured in a complete medium (Cyagen, Guangzhou, China). BMSCs were cultivated separately in either the medium alone or with various USPIO concentrations, with or without EMF exposure. Mouse primary chondrocytes were acquired from the Procell Cell Resource Center (Wuhan, China) and cultured at the Roswell Park Memorial Institute-1640 (Procell, Wuhan, China). Normal chondrocytes were cultivated in a basal medium supplemented with 10% FBS (SORFA Life Science, China). Chondrocytes in the OA conditions were treated with IL-1β (10 ng/mL) (R&D Systems, Abingdon, UK) for 24 h.

### Preparation and characterization of USPIO

The preparation, surface modification, and characterization of USPIO have been previously described [23]. USPIO was prepared using a coprecipitation technique. FeCl2 FeCl2·4H2O and FeCl3·6H2O were dissolved in 200 mL of double-distilled water (ddH2O), with ultrasonic treatment to expedite dissolution. Subsequently, 20 mL of aqueous ammonia solution (0.1 mol/L) was gradually added to the mixture while stirring for 30 min to achieve a homogeneous solution. Finally, the precipitated USPIO was rinsed with ddH2O and air-dried at 40 °C overnight.

The evaluation of USPIO was conducted with a scanning electron microscope (SEM) from Zeiss, Germany, called Merlin. The SEM was equipped with an energy-dispersive X-ray spectrometer (EDX), which was utilized for elemental mapping as well. For each sample, the dimensions and zeta potentials of the USPIO were measured three times using a Zetasizer Nano ZS instrument (Malvern, Worcestershire, UK).The TEM-2010 (Jeol, Tokyo, Japan) was used to analyze the dimensions and shape of USPIO using transmission electron microscopy (TEM). We applied 10 µL of the USPIO suspension to carbon-coated copper grids for TEM analysis.

### Electromagnetic field apparatus

The electromagnetic field-generating device was created by the Naval University of Engineering in Wuhan, China. The EMF system comprised a signal generator for producing an electromagnetic wave, an amplifier for boosting the generated signal, and a collection of Helmholtz coils. To confirm the frequency and strength of the produced EMF, an oscilloscope was employed.

### Extracellular vesicles identification and characterization

TEM was utilized for the morphological assessment of sEVs. Size distribution and quantification of sEVs were precisely determined using Nanoparticle Tracking Analysis (NTA). Furthermore, specific sEVs surface markers were validated via western blotting, complemented by nano-flow cytometry (N30 NanoFCM, Xiamen, China), which also quantified the relative abundance of these markers. The integration of NTA and nano-flow cytometry facilitates a comprehensive evaluation of sEVs’ quantity properties.

### qRT-PCR assay

TRIzol (Invitrogen, Carlsbad, CA, USA) was utilized for the extraction of cellular RNA, while the Exosome RNA Purification Kit (Umibio, Shanghai, China) was employed for the purification of miRNAs from sEVs. GAPDH and U6 served as normalization factors for mRNA and miRNA expression, correspondingly. The PCR primer sequences can be found in Supplementary Tables 2 and 3.

### Western blotting

The proteins were isolated through 10% SDS-PAGE, then transferred onto PVDF membranes, and examined with the appropriate primary antibodies. Secondary antibodies were used for blot detection. Primary antibodies were sourced from Abcam (CD9, CD63, CD81, TSG101, and Calnexin) or Cell Signaling Technology (MMP13, ADAMTS5, Aggrecan, COL 2, CD86, CD206, MFG-E8, p-P65, P65, p-IkB, and GAPDH).

### Flow cytometry analysis

CD206-APC and CD86-PE (eBioscience) were used to stain the cells.The FACSAria flow cytometer (BD Bioscience) was utilized for flow cytometry, and the data were analyzed with FlowJo V10.8.1 software.

### An in vitro chondrocyte model mimicking OA

All animal procedures were approved by the Animal Care and Use Committee of Huazhong University of Science and Technology (No.: TJH-202,103,005). Male C57BL/6 mice, aged 68 weeks, were subjected to either sham surgery or anterior cruciate ligament transection (ACLT). They were then divided randomly into six equal groups: (1) Sham (as healthy control); (2) ACLT; (3) NC-sEVs (1 × 1010 particles/mL); (4) USPIO-sEVs (1 × 1010 particles/mL); (5) EMF-sEVs (1 × 1010 particles/mL); (6) EMF-USPIO-sEVs (1 × 1010 particles/mL) groups.

### Micro-CT analysis

Micro-CT was utilized to fix and analyze the knee joint samples. 3D-MED 3.0 was employed to create reconstructed images in three dimensions. After analyzing the total volume, the area of interest included all bone spurs.

### Histological and immunofluorescence analysis

Morphological analysis involved the utilization of Hematoxylin and eosin (HE), Safranin O-fast green (SO-FG), and toluidine blue (TB) staining. For immunohistochemical examination, the sections underwent rehydration, blocking, and incubation with primary antibodies against aggrecan or MMP13 (1:100; Abcam). After being incubated with a secondary antibody (1:250; Abcam), the sections that were stained were observed using a DAB substrate and then counterstained with hematoxylin. An expert in tissue analysis, who was unaware of the research, reviewed all the pictures.

### miRNA sequencing and bioinformatics analysis

MiRNA sequencing was used to compare the miRNA expression profiles of NC-sEVs and EMF-USPIO-sEVs. miRDB, RNA22, and miRWalk were utilized to predict the potential target genes of the miRNAs. The pathways associated with osteoarthritis were investigated through Kyoto Encyclopedia of Genes and Genomes (KEGG) pathway and Gene Ontology (GO) enrichment analyses.

### Luciferase reporter assay

Wild-type (wt) and mutant (mut) 3′-UTRs of MFG-E8 and miR-99b-5p mimics or corresponding control mimics were introduced into HEK293 cells through co-transfection. The luciferase reporter system was used to assess the relative luciferase activity after 48 h.

### Statistical analysis

There was a minimum of three replicates in each experimental group. To compare disparities between two groups, the Student’s t-test was employed. The significance is denoted by * or # for p-values < 0.05, ** or ## for p-values < 0.01, and *** or ### for p-values < 0.001.

### Electronic supplementary material

Below is the link to the electronic supplementary material.

**Supplementary Material 1:** Weight percent (wt%) composition of each component in USPIO

**Supplementary Material 2:** List of mRNA primers used in this study

**Supplementary Material 3:** List of miRNA primers used in this study

**Supplementary Material 4:** Scanning Electron Microscopy-Energy Dispersive X-ray (SEM-EDX) spectroscopy mapping results for USPIO

**Supplementary Material 5:** Semi-quantitative analysis of the counts of small extracellular vesicles (sEVs): CD9-positive sEVs (A), CD63-positive sEVs (B), CD81-positive sEVs (C).

**Supplementary Material 6:** Nano-flow cytometry analysis results for small extracellular vesicles (sEVs)

**Supplementary Material 7:** Semi-quantitative fluorescence intensity results: Collagen II (COL2) Mean Fluorescence Intensity (MFI) (A), Matrix Metallopeptidase 13 (MMP13) MFI (B), CD86-positive cells (C), CD206-positive cells (D), CD86 MFI (E), and CD206 MFI (F)

**Supplementary Material 8:** Semi-quantitative assessment of: Osteoarthritis Research Society International (OARSI) grade (A), osteophyte formation scores (B), Aggrecan-positive cell count (C), and MMP13-positive cell count (D)

## Data Availability

No datasets were generated or analysed during the current study.
