# Supplementary material for: A novel extracellular vesicles production system harnessing matrix homeostasis and macrophage reprogramming mitigates osteoarthritis
Source: J Nanobiotechnology. 2024 Feb 28;22:79. doi: 10.1186/s12951-024-02324-8 (PMC10903078; doi:10.1186/s12951-024-02324-8)
Supplement: Supplementary file 1 — Supplementary Material 1: Weight percent (wt%) composition of each component in USPIO [file 12951_2024_2324_MOESM1_ESM.docx]

| Label | of total (wt %) |
| --- | --- |
| C | 39.68 |
| N | 28.31 |
| O | 25.48 |
| Ti | 0.76 |
| Fe | 5.77 |
| Total | 100 |

Table S1. wt% content of each component for USPIO
