# Supplementary material for: A novel extracellular vesicles production system harnessing matrix homeostasis and macrophage reprogramming mitigates osteoarthritis
Source: J Nanobiotechnology. 2024 Feb 28;22:79. doi: 10.1186/s12951-024-02324-8 (PMC10903078; doi:10.1186/s12951-024-02324-8)
Supplement: Supplementary file 2 — Supplementary Material 2: List of mRNA primers used in this study [file 12951_2024_2324_MOESM2_ESM.docx]

Table S2. The mRNAs primer utilized in this study

| Gene | Primer Sequence, 5’–3’ | |
| --- | --- | --- |
|  | Forward | Reverse |
| *MMP13*  *ADAMTS5*  *Aggrecan*  *COL 2*  *GAPDH*  *U6* | TGTTTGCAGAGCACTACTTGAA  CCCAGGATAAAACCAGGCAG  GTGGAGCCGTGTTTCCAAG  GGGTCACAGAGGTTACCCAG  AGGTCGGTGTGAACGGATTTG  TGGAACGCTTCACGAATTTGCG | CAGTCACCTCTAAGCCAAAGAAA  CGGCCAAGGGTTGTAAATGG  AGATGCTGTTGACTCGAACCT  ACCAGGGGAACCACTCTCAC  GGGGTCGTTGATGGCAACA  GGAACGATACAGAGAAGATTAGC |
