# Supplementary material for: A novel extracellular vesicles production system harnessing matrix homeostasis and macrophage reprogramming mitigates osteoarthritis
Source: J Nanobiotechnology. 2024 Feb 28;22:79. doi: 10.1186/s12951-024-02324-8 (PMC10903078; doi:10.1186/s12951-024-02324-8)
Supplement: Supplementary file 3 — Supplementary Material 3: List of miRNA primers used in this study [file 12951_2024_2324_MOESM3_ESM.docx]

Table S3. The miRNAs primer utilized in this study

| miRNA | Primer sequence, 5’–3’ |
| --- | --- |
| *miR-99b-5p mimics*  *miR-99b-5p inhibitor*  *mimics NC*  *inhibitor NC* | CACCCGUAGAACCGACCUUGCG  CGCAAGGUCGGUUCUACGGGUG  UUCUUCGAACGUGUCACGUUU  CAGUACUUUUGUGUAGUACAA |

Figure S1: BMSC-Fe_3_O_4_-SMF-Exo-secreted miR-1260a can be transferred to BMSCs and HUVECs via exosomes. (***) *p* < 0.001, ns = no significance. (a, b) Levels of mature and pri/pre miR-1260a in exosome-treated BMSCs and HUVECs. (c) Treatment with an RNA polymerase II inhibitor did not alter the level of miR-1260a in BMSCs or HUVECs exposed to BMSC-Fe_3_O_4_-SMF-Exos.
