# Supplementary figures and images for: A novel extracellular vesicles production system harnessing matrix homeostasis and macrophage reprogramming mitigates osteoarthritis
Source: J Nanobiotechnology. 2024 Feb 28;22:79. doi: 10.1186/s12951-024-02324-8 (PMC10903078; doi:10.1186/s12951-024-02324-8)

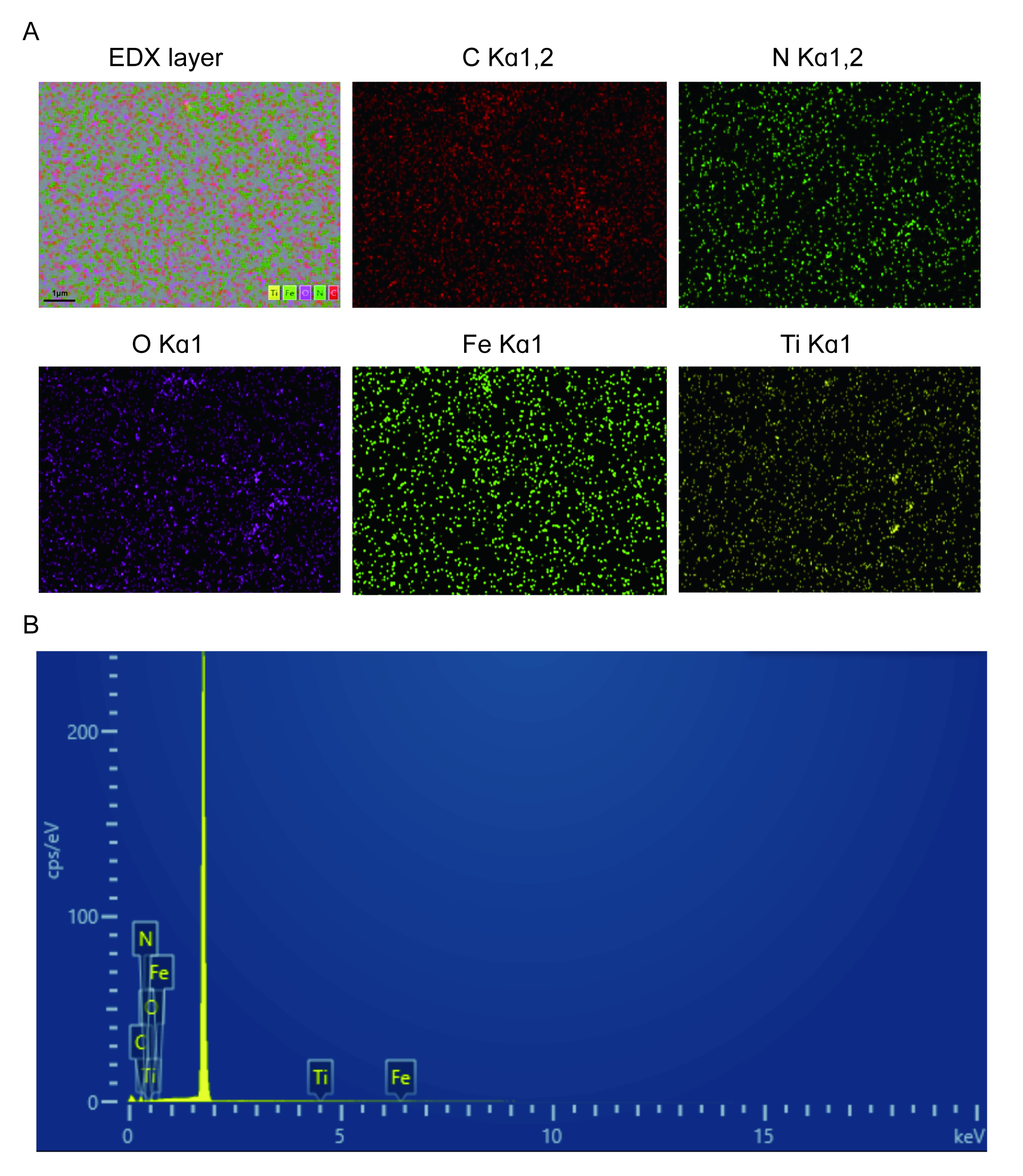

Supplement: Supplementary file 4 — Supplementary Material 4: Scanning Electron Microscopy-Energy Dispersive X-ray (SEM-EDX) spectroscopy mapping results for USPIO [file 12951_2024_2324_MOESM4_ESM.tif]

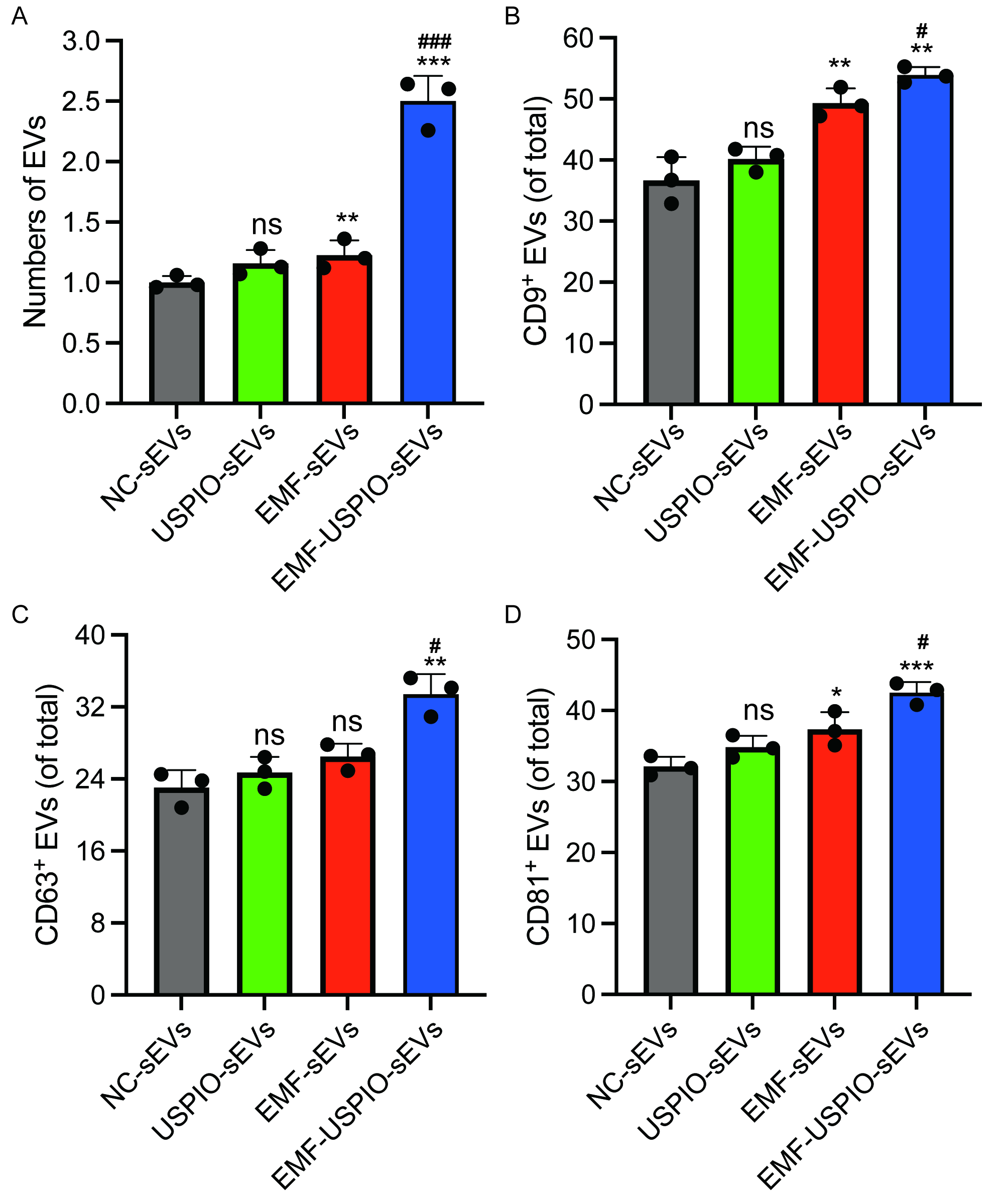

Supplement: Supplementary file 5 — Supplementary Material 5: Semi-quantitative analysis of the counts of small extracellular vesicles (sEVs): CD9-positive sEVs (A), CD63-positive sEVs (B), CD81-positive sEVs (C). [file 12951_2024_2324_MOESM5_ESM.tif]

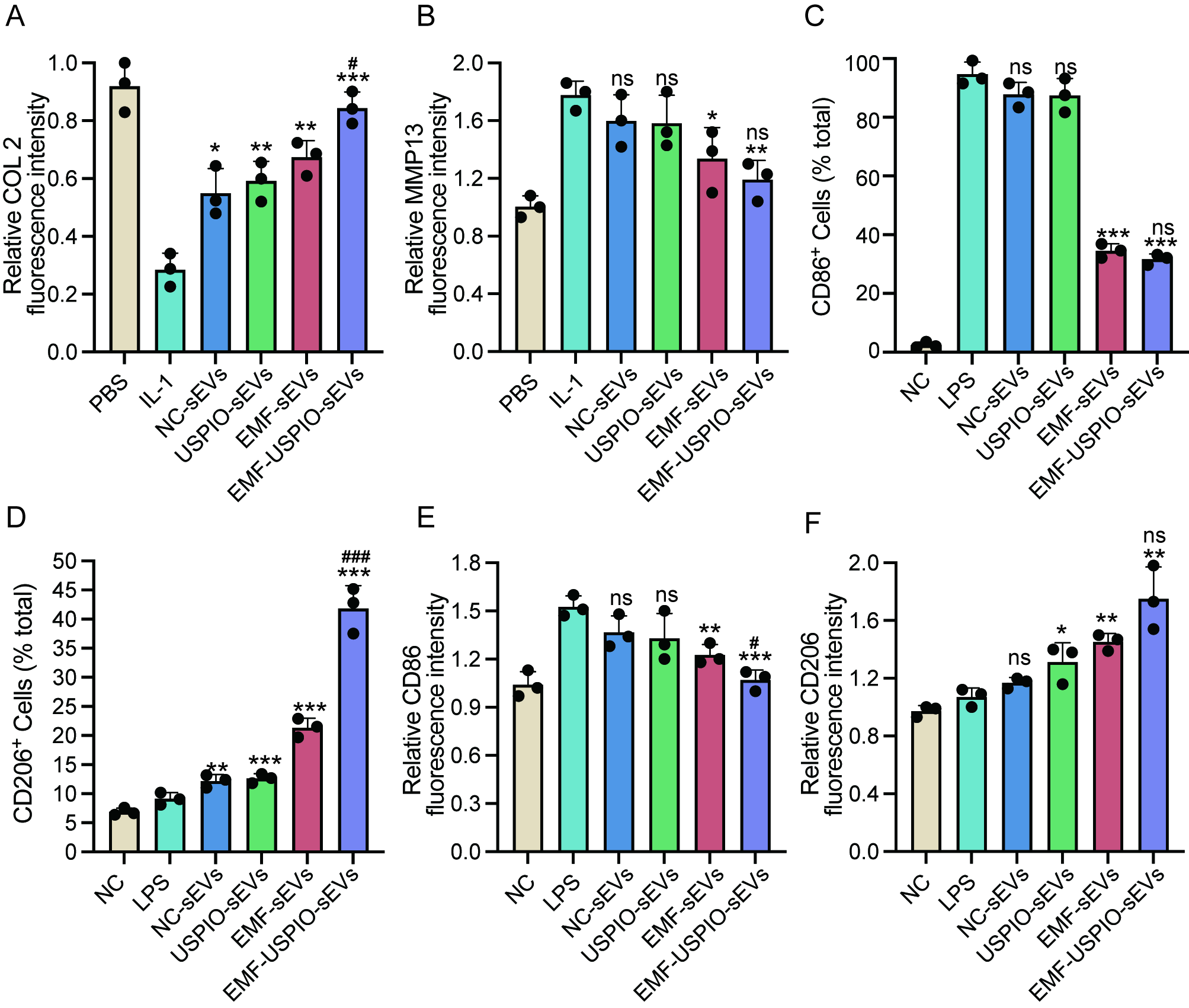

Supplement: Supplementary file 6 — Supplementary Material 6: Nano-flow cytometry analysis results for small extracellular vesicles (sEVs) [file 12951_2024_2324_MOESM6_ESM.tif]

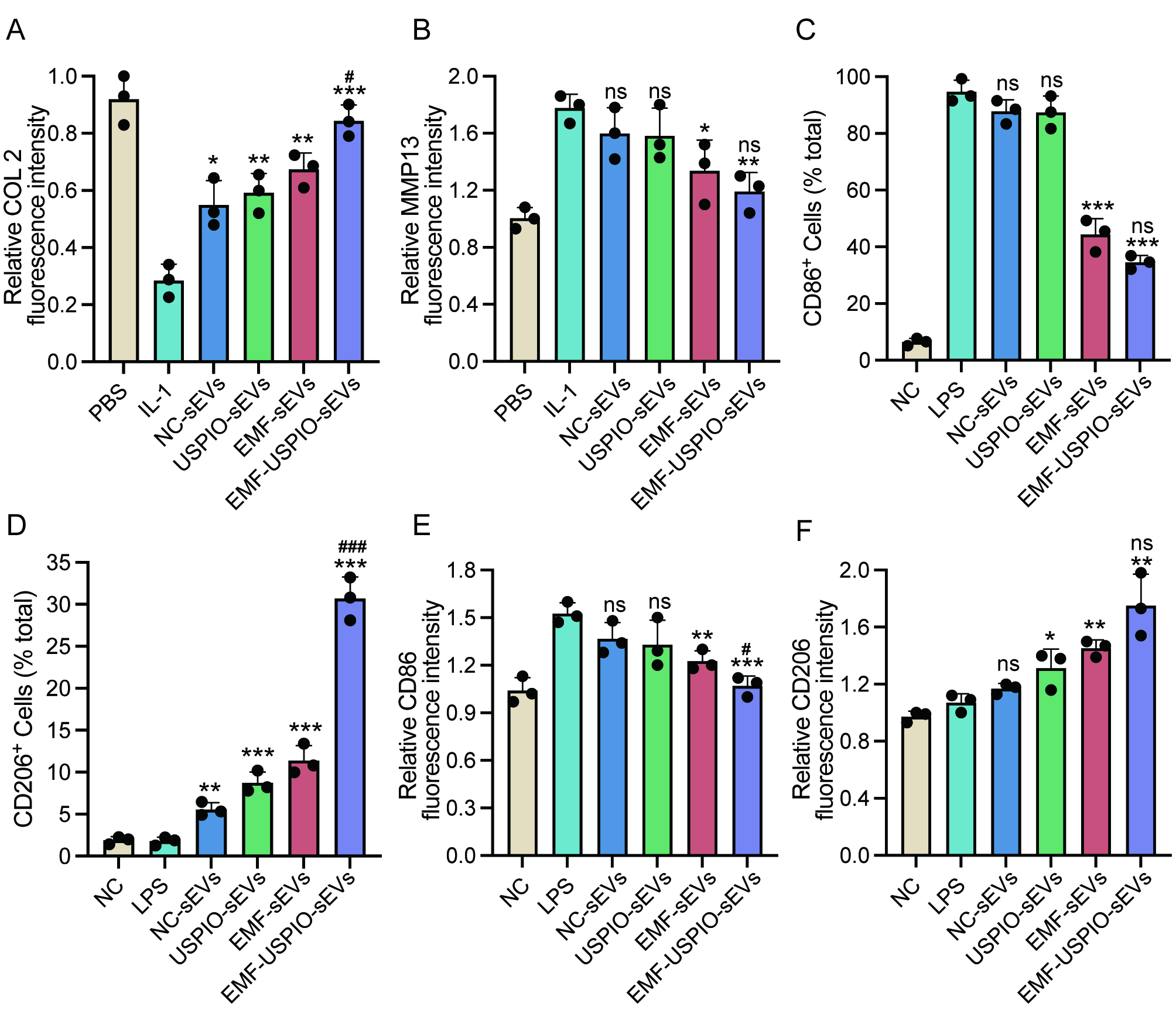

Supplement: Supplementary file 7 — Supplementary Material 7: Semi-quantitative fluorescence intensity results: Collagen II (COL2) Mean Fluorescence Intensity (MFI) (A), Matrix Metallopeptidase 13 (MMP13) MFI (B), CD86-positive cells (C), CD206-positive cells (D), CD86 MFI (E), and CD206 MFI (F) [file 12951_2024_2324_MOESM7_ESM.tif]

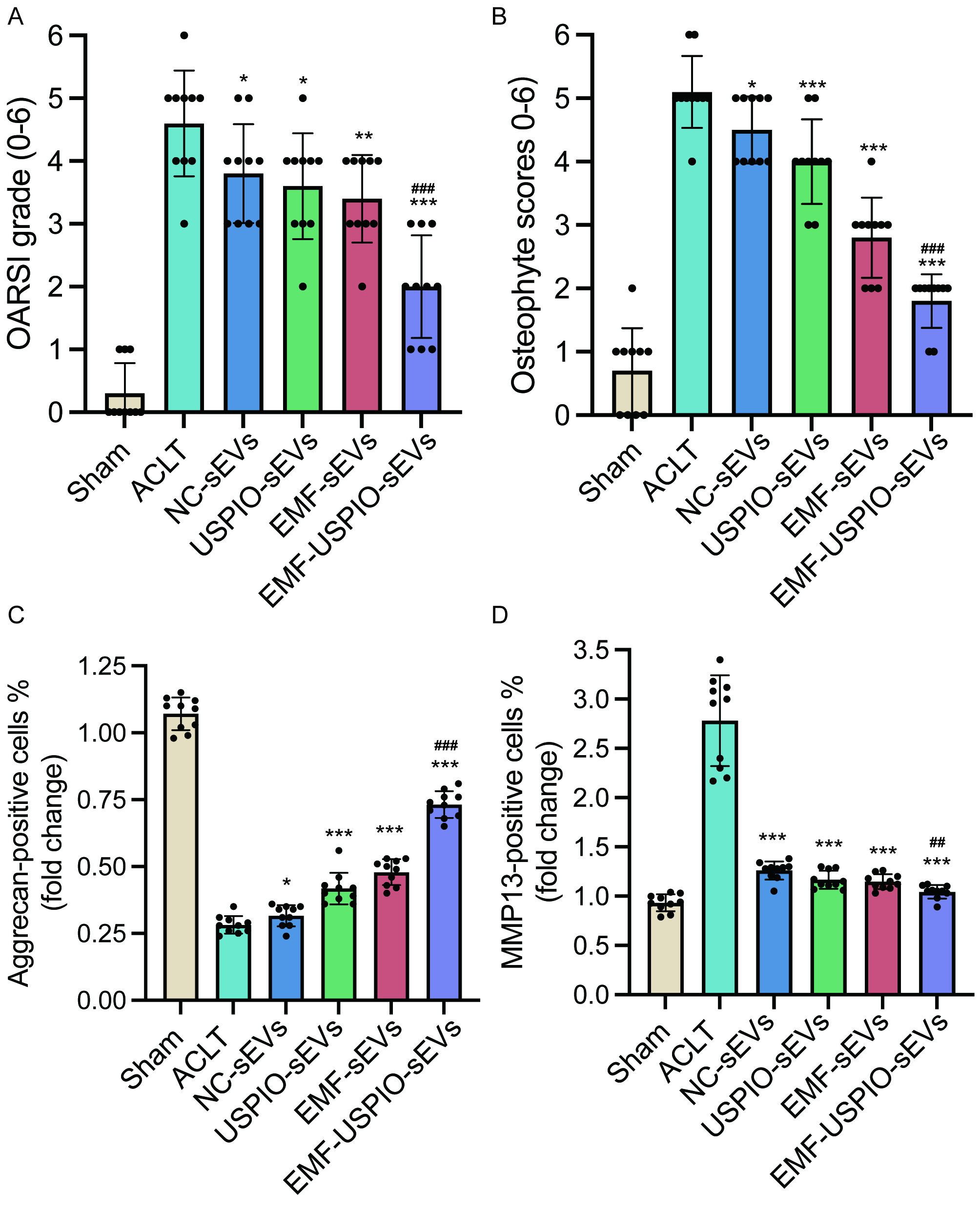

Supplement: Supplementary file 8 — Supplementary Material 8: Semi-quantitative assessment of: Osteoarthritis Research Society International (OARSI) grade (A), osteophyte formation scores (B), Aggrecan-positive cell count (C), and MMP13-positive cell count (D) [file 12951_2024_2324_MOESM8_ESM.tif]
